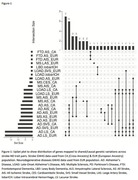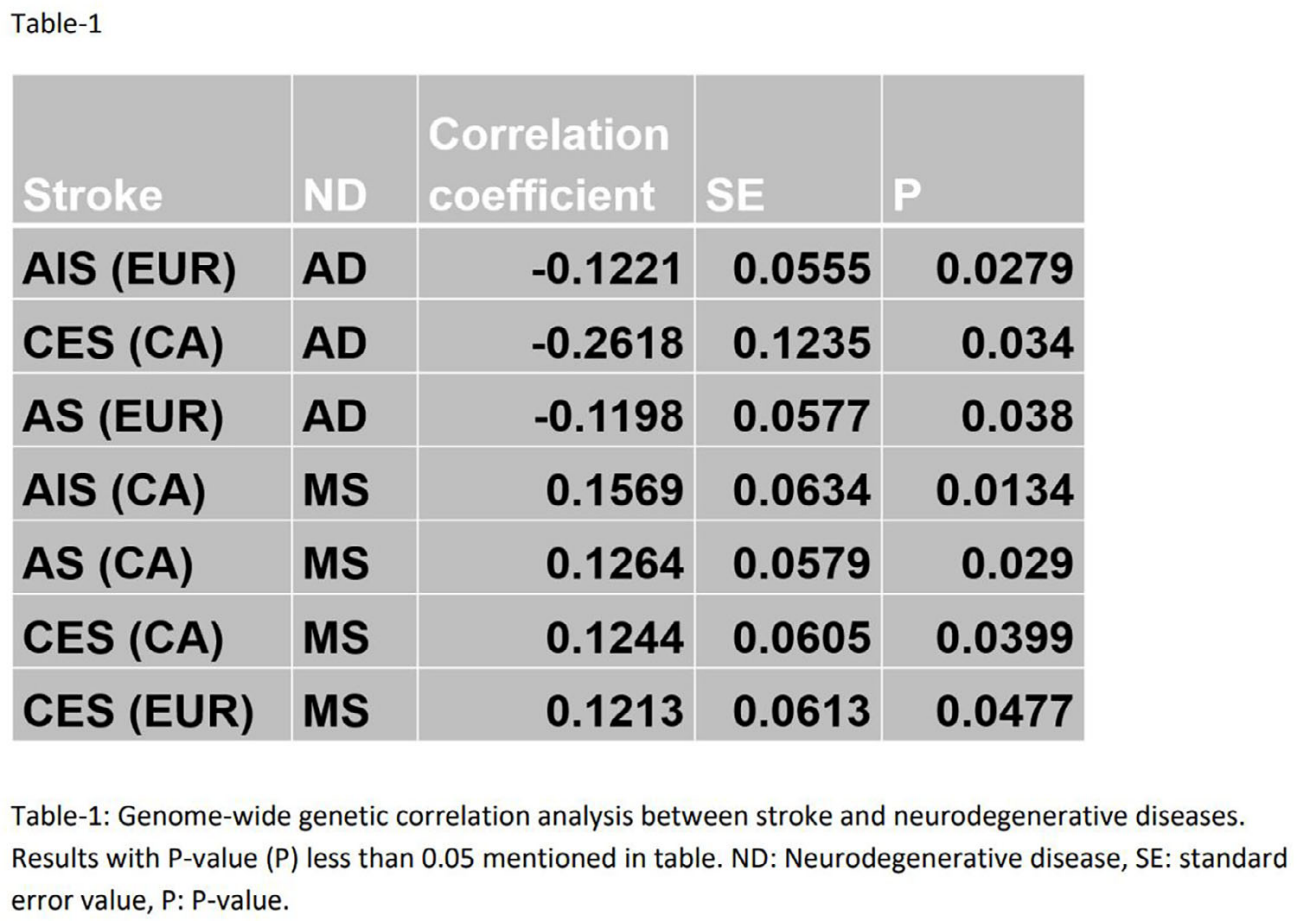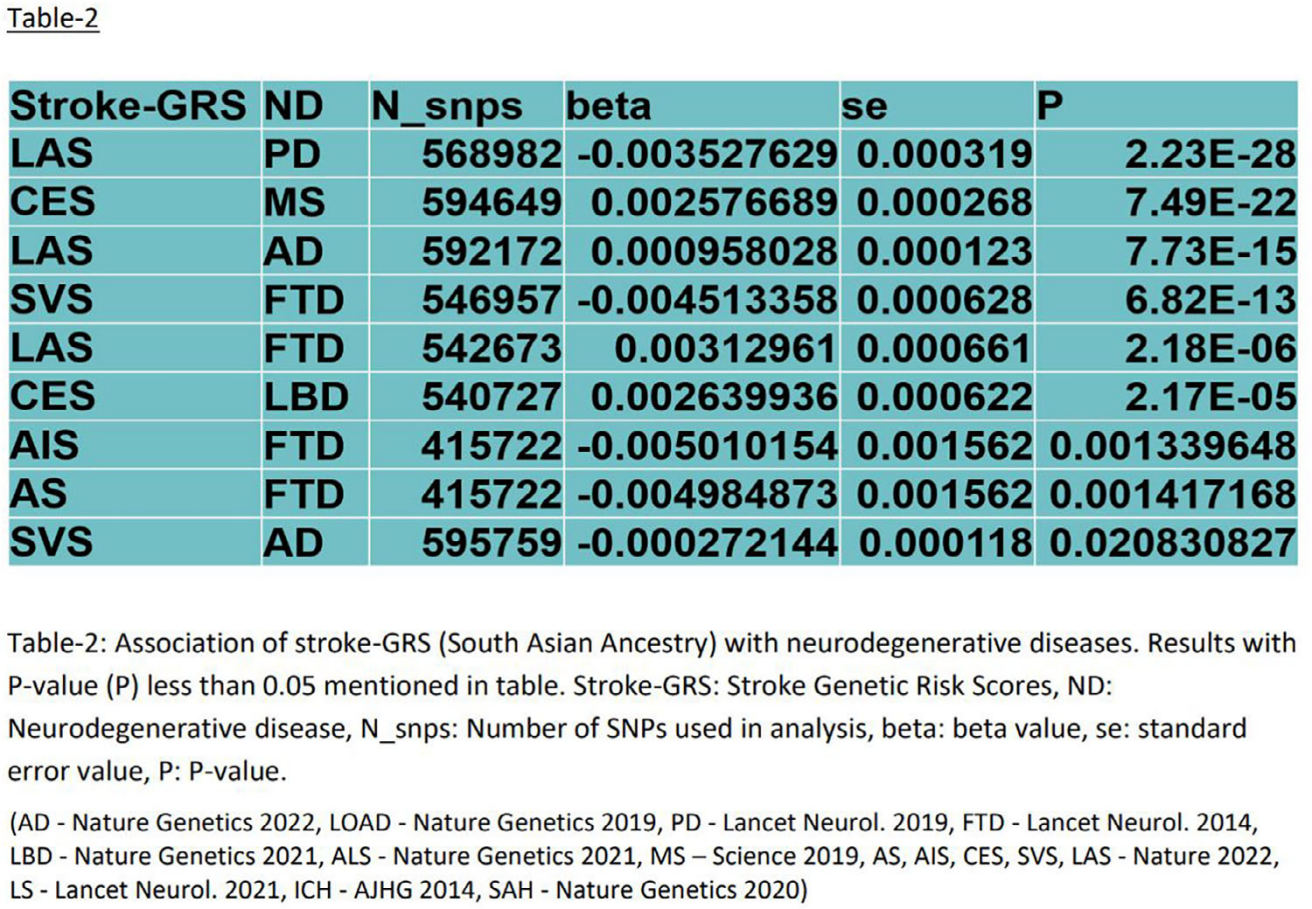# Large‐scale discovery of novel shared genetic etiology between stroke and neurodegenerative diseases like Dementia, Multiple sclerosis, Parkinson’s Disease, Amyotrophic Lateral Sclerosis

**DOI:** 10.1002/alz.090140

**Published:** 2025-01-03

**Authors:** Akhilesh Shailendra Khamkar, Ganesh Chauhan

**Affiliations:** ^1^ Rajendra Institute of Medical Sciences (RIMS), Ranchi, Jharkhand India; ^2^ Department of Genetics and Genomics, Rajendra Institute of Medical Sciences, Ranchi, Jharkhand India

## Abstract

**Background:**

Stroke, a cerebrovascular condition, and neurodegenerative diseases (ND) like Dementia, Multiple sclerosis, Parkinson’s Disease, Amyotrophic Lateral Sclerosis are major types of neurological disorders, which are associated with increasing global morbidity and mortality burden. But to what extent shared genetic architecture is involved between stroke and ND is unknown.

**Method:**

We investigated shared genetics between stroke (10 subtypes) and ND (6 diseases) using large scale Genome‐Wide Association Study (GWAS) summary statistics data for Cross‐Ancestry, European and South Asian samples including Indians. We used largest GWAS results for AD from EADB and for stroke subtypes from GIGASTROKE consortium. Different computational approaches ‐ LDSC, PLACO, COLOC was used to assess genetic overlap between stroke and ND. Functional annotation and gene‐set enrichment analysis (FUMA) was executed to identify genes and molecular pathways to get detailed insight into shared disease mechanisms between stroke and ND. We performed genetic risk score (GRS) analysis to estimate combined effect of stroke associated genetic variants on ND.

**Result:**

We found negative genetic correlation between Alzheimer’s disease and stroke subtypes (all, ischemic & cardioembolic) at genome‐wide level (correlation coefficient (rg): ‐0.12 to ‐0.26, P value: 0.028 to 0.038) (Table‐1). 23 genetic variations showed association with ND (Alzheimer’s disease, Multiple sclerosis) and stroke subtypes (lacunar, ischemic) at genome‐wide significance level (P<5 × 10^‐8^). We found 184 causal lead pleiotropic SNPs for stroke‐ND trait pairs (Figure‐1). Biological pathway analysis shows that shared genes are highly enriched in pathways related to immune system, protein catabolism, phagocytosis. Genetic risk scores of stroke subtypes for South Asians including Indian samples shows association with neurodegenerative diseases (beta: ‐0.005 to 0.003, P value: 2.23 × 10^‐8^ to 0.021) (Table‐2).

**Conclusion:**

This is first study which shows extensive pleiotropy present between stroke and neurodegenerative diseases at genome‐wide level. We found GRS of stroke subtypes for South Asian samples also associated with neurodegenerative diseases which suggest that applicability of GIGASTROKE GWAS data of Indians in prediction of neurodegenerative diseases; despite smaller sample size compared to other ancestries in world. These research findings have strong implication for future development of early diagnosis, prevention and treatment strategies for both cerebrovascular and neurodegenerative diseases.